# Physiological Disorders and Fruit Quality Attributes in Pomegranate: Effects of Meteorological Parameters, Canopy Position and Acetylsalicylic Acid Foliar Sprays

**DOI:** 10.3389/fpls.2021.645547

**Published:** 2021-03-11

**Authors:** Pavlina Drogoudi, Georgios E. Pantelidis, Stavroula A. Vekiari

**Affiliations:** ^1^Department of Deciduous Fruit Trees, Institute of Plant Breeding and Genetic Resources, Hellenic Agricultural Organization (HAO) ‘Demeter’, Naousa, Greece; ^2^Institute of Technology of Agricultural Products, HAO ‘Demeter’, Athens, Greece

**Keywords:** anthocyanins, antioxidants, cracking, fruit weight and number, russeting, sun scald, total soluble content, yield

## Abstract

Meteorological parameters and occurrences of cracking (CR), russeting (RS), and sun scald (SS) symptoms were monitored in a pomegranate cv. “Wonderful” orchard planted in a W–E orientation, during a 3-year study. Moreover, the efficacy of preharvest foliar sprays with acetylsalicylic acid (ASA; 0.5 mM or 1.0 mM), applied biweekly four to six times, on yield and fruit quality attributes were evaluated in a 2-year study. Fruit from the N-side of the canopy had greater CR and RS, whereas SS symptoms were lower, compared with the S-exposed part of the canopy. The N-side of the canopy had also substantially lower fruit number and yield, suggesting for an important role of light on bisexual flower formation and/or fruit set. Following the occurrences in CR and RS during the fruit maturation period, it was found that temperature fluctuation was the main cause. The presence of RS damages may also be related with increased relative humidity and water movement as symptoms were higher in years with higher values, in the N-side of the canopy and often occurred in the exposed and stylar end of the fruit. The ASA treatment substantially reduced RS by up to 57%, improved the peel red coloration, while anthocyanin, antioxidant capacity, and soluble solid contents in juice were higher. Foliar sprays with ASA did not affect yield, but induced a trend of bigger-sized fruit. In conclusion, planting in a N–S row orientation and selecting an orchard plantation site with a minimum temperature fluctuation and low relative humidity during the fruit-ripening period are measures to control CR and RS in pomegranate. ASA foliar applications proved to have beneficial effects on juice antioxidant contents, but more importantly on fruit appearance.

## Introduction

The pomegranate fruit has beneficial effects on human health and very good organoleptic characteristics, which were strong arguments for its promotion in the markets of developed countries that previously did not know much about this crop. The majority of pomegranates are produced in tropical and subtropical parts of the world, but recently, there was an expansion of the cultivated areas due to a greater consumer’s demand. Pomegranate is considered to be adapted to a wide range of climates and soil conditions. In Greece, production was traditionally located in Peloponissos, southern Greece, with a dry and hot Mediterranean climate, using the local cultivar “Ermioni.” Since 2006, its cultivation expanded in northern parts of the country, with more wet and colder climate, with the introduced cultivar “Wonderful,” reaching nowadays about 3.000 ha.

Of the most important cause limiting commercial yield worldwide is cracking (CR). It is induced by differences in the growth rate between peel and flesh of the fruit and the pressure imposed by the quickly expanding arils on the stretched peel (Singh et al., 2020). Cultivars differ in their susceptibility to CR and damages were related with increasing trends in fruit volume, lower Ca ion and higher pectin content in peel, resulting in decreased elasticity (Saei Ahagh et al., 2015). Little is known on the magnitude of the influence caused by changes in environmental parameters on CR on pomegranate or other fruits (Khadivi-Khub, 2015). Rainfall in previously water stressed pomegranate trees induced CR due to an asymmetric increase in fruit turgor pressure; aril turgor increased to a much greater extent than peel turgor (Galindo et al., 2014). In tomato, which is considered as a model system for studying cracking, irrigation together with environmental conditions such as high relative humidity (RH) and radiation that modify the growth rate during ripening also favored fruit cracking (Guichard et al., 2001; Domínguez et al., 2012). Symptoms of RS in pomegranate appear as tiny cracks in the cuticle and corky like surface in the peel; being restricted in only a portion of fruit located mainly in the sides or lower part of the fruit and in some cases covering its surface (Supplementary Photo 1). There is little documentation on **RS damages** and its causal agents in pomegranate. Russeting is a common physiological disorder in apples, pear, and plums that develops in only some cultivars and occur by stress like unfavorable weather conditions, especially low temperatures in fruit setting, pesticide application at high temperatures, or incompatible pesticide mixtures (Tukey, 1959; Michailides, 1991; Khanal et al., 2013). RS damages may be substantial in cv. “Wonderful,” affecting up to 70% of fruit in some orchards and years in Northern Greece. We have not seen RS damages in fruit from the fully red colored cv. “Acco.”

In recent years a significant number of studies have documented that exogenous **salicylic acid** act as an effective “therapeutic agent” for plants just like it is for mammals, such as by developing resistance to abiotic stress conditions or providing protection against pathogens (Koo et al., 2020). Acetyl salicylic acid (ASA) is a close analog of salicylic acid and when applied exogenously is converted to salicylic acid spontaneously. Besides this function during biotic and abiotic stress, salicylic acid plays a crucial role in the regulation of physiological and biochemical processes during the entire lifespan of the plant, by affecting a wide range of processes, such as seed germination, photosynthetic processes, growth, flowering, fruit ripening, and others (Rivas-San Vicente and Plasencia, 2011; Koo et al., 2020). However, the above effects of salicylic acid are not always constant depending on the method of application, plant species, the plant developmental stage, and the concentration used (Horváth et al., 2007; Rivas-San Vicente and Plasencia, 2011).

Preharvest foliar sprayings with salicylic or ASA induced a range of beneficial effects such as improvement in fruit size, bioactive compounds, sugars, and organic acids in fruit trees species such as apples (Shaaban et al., 2011; Giannousis, 2012), pears (Cao et al., 2006), sweet cherries (Yao and Tian, 2005; Giménez et al., 2014, 2017; Valverde et al., 2015), oranges (Huang et al., 2008), grapes (Champa et al., 2015; Oraei et al., 2019; García-Pastor et al., 2020b), and plums (Martínez-Esplá et al., 2017, 2018). However information is scarce for pomegranate and especially for the widely grown cv “Wonderful,” being a monoculture for the United States (Chater et al., 2018) and the main cultivar introduced in Greece, Cyprus, and other countries. In the study by Ahmed et al. (2014), application of salicylic acid at 100 ppm, together with various nutrients, reduced CR symptoms in cv. “Manfalouty,” but there was no effect when salicylic acid or nutrients were applied separately. In a recent study, preharvest salicylic acid, ASA, and methylsalicylic acid foliar applications on pomegranate improved the fruits’ quality characteristics and its nutritional status on pomegranate cv. “Mollar de Elche” (García-Pastor et al., 2020a). When applied after harvest in pomegranate fruit cv. “Mollar de Elche,” salicylic acid, or ASA treatments reduced chilling injury during storage (Sayyari et al., 2011b; Sayyari and Valero, 2012).

The aims of the present study were to investigate the effects of: (i) meteorological parameters, (ii) fruit position in the canopy, and (iii) preharvest ASA applications, on the occurrence of physiological disorders, yield parameters, fruit quality, and antioxidant attributes in a commercial pomegranate cv. “Wonderful” orchard during a 3-year study. The study was facilitated with the selection of an experimental orchard that intense symptoms of physiological disorders such as CR and RS were regularly monitored. There is scarce information on the effects of environmental and cultivation parameters on the presence of physiological parameters in pomegranate as well as effects of ASA on cv. “Wonderful.”

## Materials and Methods

The experiments were conducted in a commercial pomegranate (*Punica granatum* cv “Wonderful”) orchard located in Neochori Imathias, central Macedonia, Greece (40°40’15.49” B, 22°27’53.94” E, 4 masl, Mediterranean temperate climate). The trees were 5 years old, trained in a tree shape, supported with trellises, and planted at 6 × 2 m spacing, in west-east rows. All trees received routine horticultural care.

### Monitoring the Occurrence of Physiological Disorders in Different Years and Sides of Canopy

Symptoms of CR, RS, and SS were monitored during the fruit maturation period, in 2011–2013. Measurements were made in eight trees selected for crop load, shape, and size uniformity in a complete randomized design with four replicates and each replicate was represented by two trees. The number of fruit without any defect and those with CR, RS, or SS symptoms were counted in the north (N-) and south (S-) side of canopy, being separated by the presence of tiers in trellises. The percentage of intact fruit and those with physiological disorders were calculated.

Temperature was recorded hourly using a “SKY data hog” (Skye instruments, Powys, United Kingdom), positioned in the experimental orchard at about 1.5 m height, and daily maximum (T_*max*_) and minimum (T_*min*_) temperatures were calculated. Percentage (%) relative humidity (%RH) and rainfall data were used from a meteorological station located at the Department of Deciduous Fruit Trees in Naousa, where measurements were taken using an on-site meteorological station (46703 portable hygrothermograph, Forli, Italy, and standard rain-gauge, respectively).

### Acetylsalicylic Acid (ASA) Treatments

Twenty-four trees were selected for crop vegetative and crop load uniformity at random within the orchard. The experiment was designed as randomized complete block design with four replicates for each treatment and each replicate was represented by two trees. Foliar sprays of 0.5 mM ASA or 1.0 mM ASA were carried out biweekly starting on 230 (August 19), 244, 258, and 272 days in the year 2011, and on 198 (July 17), 211, 225, 240, 253, and 267 days in the year 2012. The last foliar application was 15 days before commercial harvest in both years. Control trees were left unsprayed. Buffer trees were used within the row to minimize the spray drift between treatments. The surfactant Shinulin (Farma Chem SA, Thessaloniki, Greece) was added at the rate of 0.013% in all sprayings in order to obtain best penetration results. Foliar treatments were applied until the point of runoff between 8h00 and 11h00, using a petrol engine sprayer. At the beginning of spraying with ASA there were no symptoms of CR, SS, or RS on the pomegranate fruits.

#### Yield, Physiological Disorders, and Fruit Physical Characteristics

Two days before the first commercial harvest the number of fruits without any defect, CR, RS, and SS symptoms were counted separately in the N- and S-exposed part of the canopy and the percentage of fruit with physiological disorders were calculated. The fruits were harvested in two harvest dates following the producer criteria, including the attainment of 16 °Brix, size > 350 g and peel color intensity and distribution. The mean fruit fresh weight (g), percentage fruit of different weight categories, mean fruit number tree^–1^, and yield (kg tree^–1^) were calculated. Yield measurements were made at two harvest dates only in 2012.

A total of 16 fruits harvested from the S-side of the canopy, one from each replicate tree, of similar size, external color, and without any defects were separated at the second commercial harvest, transferred to the laboratory where the fruits’ physical measurements and samplings for the chemical measurements were made within 24 h.

Each fruit was weighted and intact arils were separated by hand from the pith and carpellary membranes (*n* = 6). One hundred arils were weighted. The peel and aril fresh weight was determined. Juice was extracted from the isolated arils by hand squeezing, weighted, and the percentage (%) juice content was calculated.

The CIE color parameters *L*^∗^ (brightness or lightness; 0 = black, 100 = white), a^∗^ (-a^∗^ = greenness, + a^∗^ = redness) and b^∗^ (-b^∗^ = blueness, + b^∗^ = yellowness), hue (H^∗^) (calculated as tan^–1^b^∗^/a^∗^; 0° = red-purple, 90° = yellow, 180° = bluish-green, 270° = blue) and Chroma (C^∗^) (calculated as (a^*2^+ b^*2^)^1/2^; degree of departure from gray to pure chromatic color) were measured in 12 fruit replicates, using a Minolta chromatometer (Minolta CR-300, Ramsey, NJ). Readings were taken in the sun exposed part of each fruit exocarp in the year 2012.

#### Soluble Solid and Total Acid Contents

In freshly extracted juice the soluble solid content (SSC) and titratable acidity (TA) were determined in six replicate fruits. SSC was measured using a digital refractometer (model PR-1, Atago, Japan) and the data were expressed as °Brix. TA was measured by titrating 5 ml of juice with 0.1 N NaOH to a pH end point of 8.2. Results were expressed as g citric acid equivalent L^–1^. Maturity index (MI) was calculated as the SSC/TA ratio.

#### Total Phenols

The total phenol (TPs) content was determined using the Folin–Ciocalteu colorimetric method (Singleton and Orthofer, 1999). The reaction mixture consisted of 0.3 ml of diluted extract, 0.2 ml of distilled water, and 2.5 ml of 10% Folin–Ciocalteu reagent. The tube was vortexed and then allowed to stand at room temperature for 3 min when 2 ml of saturated sodium carbonate solution was added. The solution was incubated for 5 min at 50°C, and the absorbance was measured at 760 nm against a blank solution. Each measurement was repeated in duplicate. The total phenolic content was expressed as mg gallic acid equivalents L^–1^.

#### Total Antioxidant Capacity

Total antioxidant capacity was evaluated using the 1,1-diphenyl-2-picryl hydrazyl (DPPH) (TAC_*DPPH*_) assay. The reaction mixtures containing 0 or 20 μl of diluted juice (1:5 in MeOH), 2.3 ml of 106.5 μM DPPH in MeOH, and 680 μl of H_2_O were vortexed, then kept at room temperature in the darkness for 4 h (Blois, 1958). The absorbance of each reaction mixture was measured at 517 nm. A standard curve was obtained by using ascorbic acid standard solution, and accordingly, results were expressed as mg ascorbic acid equivalent L^–1^.

#### Ascorbic Acid

Ascorbic acid was determined using the reflectometer Merck RQflex. Two milliliters of pomegranate juice was diluted with 3 ml of ice-cold 6% (w/v) metaphosphoric acid containing 0.2 mM ETDA. Approximately 500 mg polyvinylpolypyrrrolidone Divegran RS to 10 ml of the diluted juice, and pH was adjusted to <1 using sulfuric acid. The sample was mixed for approximately 1 min, the extract was centrifuged at 15,000 × *g* for 7 min at 4°C, and the ascorbate content was measured in the supernatant. The concentration of ascorbic acid in the sample was quantified using a standard curve of known concentration of l-ascorbic acid (Sigma) and the final results were expressed as mg ascorbic acid equivalent L^–1^.

#### Organic Acids

Organic acids were identified and quantified by HPLC according to the method described by Mikulic-Petkovsek et al. (2012). The samples were analyzed with a Hewlett Packard, Agilent series 1100 HPLC liquid chromatograph equipped with a G1311AQuat Pump. An MZ-LiChrospher RP 18/5 μm type analytical column (250 × 4.6 mm) and a 20-μl loop injector were used. The mobile phase was HPLC-grade water/metaphosphoric acid (until the pH was 2.2) at a flow rate of 1.1 ml min^–1^. The detector wavelength was fixed at 245 nm.

#### Anthocyanin and Elagic Acid

Analyses of anthocyanins and ellagic acid were performed according to the method described by Mousavinejad et al. (2009) by HPLC. A C18 MZ-Analytical Hypersil ODS 5μm column 250 × 4.6 mm (ser. No 15210315) was used for the separation of sample components. Mobile phase consisted of solvent A (2.5% v/v, solution of acetic acid in methanol) at different ratios, the gradient profile was 100% A at 0–5 min, 90% A at 15 min, 50% A at 45 min, and 100% A at 55 min. Flow rate was 1.0 ml min^–1^. Chromatograms were recorded at 510 nm. Due to similarity with anthocyanins, ellagic acid was eluted immediately after anthocyanins on the chromatograms. A standard library was constructed from delphinidin 3,5-diglucoside (Dp3,5), cyanidin 3,5-diglucoside (Cy3,5), delphinidin 3-glucoside (Dp3), and cyanidin 3-glucoside (Cy3). Peaks for delphinidin 3,5-diglucoside (Dp3,5), cyanidin 3,5-diglucoside (Cy3,5), delphinidin 3-glucoside (Dp3), and cyanidin 3-glucoside (Cy3) were identified and quantified with standards, using a data processing system, and expressed on a fresh weight (FW) basis.

### Statistical Analyses

Means ± SE were calculated. Statistical analyses were carried out using a multi-factor ANOVA, using ASA, canopy side and year as factors, based upon the replicate tree or fruit. Results on the presence of physiological disorders on different Julian dates were subject to one-way ANOVA. Treatment means were separated using a Duncan multiple range test where ANOVA *F-*tests were significant at *p* < 0.05 variance. Pearson correlation analyses were performed between changes in temperature and changes in records of physiological disorders. Percentage data were arcsine and ratio data were log transformed before analyses. The statistical package SPSS was used (SPSS Inc., Chicago, IL, United States).

## Results

### Occurrences of Physiological Disorders in Different Years

**FIGURE 1:**
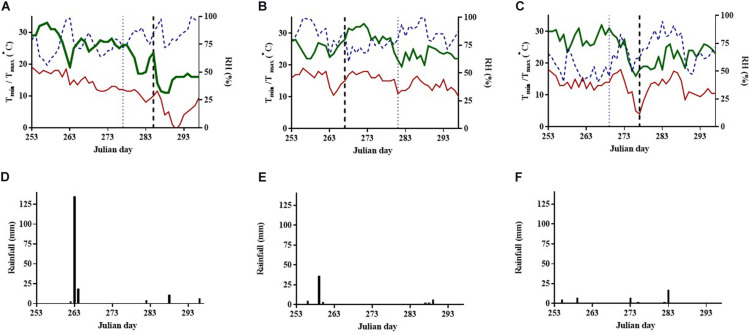
Changes in **(A–C)** daily minimum (T_*min*_, solid thin line) and maximum air temperature (T_*max*_, solid thick line) (°C), relative humidity (RH%, dotted line) and **(D–F)** daily rainfall (mm, solid bars) during the ripening periods in years **(A,D)** 2011, **(B,E)** 2012, and **(C,F)** 2013 in a pomegranate cv. ‘Wonderful’ orchard. Vertical dotted lines on x axes depicted in a-c graphs, show the timings that fruit maturation (thin dotted lines) and cracking and russeting symptoms (thick dotted lines) firstly occurred in each year.

In Figure 1, meteorological parameters such as T_*min*_, T_*max*_, %RH, and rainfall are presented during the last period of fruit maturation (253–297 days, from September 10 to October 25) in 2011–2013. Moreover the days of first CR, RS, and SS occurrence and fruit maturation are indicated.

Mean T_*max*_, mean T_*min*_, and T_*min*_ in the above studied period were higher in 2012, followed by 2013 and 2011 (mean T_*max*_, 25.7°C, 24.6°C, and 22.9°C; mean T_*min*_, 15.0°C, 12.9°C, and 11.6°C, lowest T_*min*_, 10.5°C, 4.0°C, and -0.5°C, respectively). The average %RH was higher in 2011 and 2012, compared with 2013 (79.1%, 82.3%, and 63.4%, respectively). The rainfall occurrences were similar in the three studied years (sum of 44–55 mm), apart from having one extra rainy day with 135 mm in 2011 (Figures 1D–F).

In 2011, fruit maturation initiated in 277 days and an increase in the %CR (from 2.5% to 17.0%) and %RS (from 3.1% to 13.1%) were recorded 8 days later (285 days), and coincided with a fluctuation in T_*max*_ (varied between 28.0°C and 17.0°C) and T_*min*_, and no change in %RH or rainfall (Table 1; Figure 1). A further substantial increase in the %CR (from 17.0% to 55.4%) and %RS (from 13.1% to 40.2%) symptoms was recorded on day 298, resulting from a substantial drop in temperature to -0.5°C.

**TABLE 1:** Percentage cracking (%CR), russeting (%RS), and sun-scald (%SS) (mean ± SE) and changes in CR, RS, and temperature [maximum-minimum values in daily mean maximum (T_*max*_) and minimum (T_*min*_) temperature (°C)], in the period before the measurement dates.

	2011			2012			2013	
			
	277 d	285 d	298 d	263 d	267 d	288 d	268 d	277 d
%CR (Change)	2.5 ± 1.3c	17.0 ± 3.8b (14.5%)	55.4 ± 1.6a (38.4%)	4.0 ± 1.0c	11.1 ± 1.9b (7.1%)	53.5 ± 5.0a (42.4%)	0.0 ± 0.0b	14.9 ± 2.9a (14.9)
%RS (Change)	3.1 ± 0.8c	13.1 ± 2.4b (10.0%)	40.2 ± 7.9a (27.1%)	9.0 ± 1.9b	32.9 ± 2.8b (23.9%)	43.8 ± 3.0a (10.9%)	0.0 ± 0.0b	27.6 ± 5.0a (27.6%)
%SS	20.5 ± 3.5	21.5 ± 8.3	21.5 ± 8.3	23.4 ± 1.0	23.3 ± 1.3	22.1 ± 1.5	9.9 ± 1.1	10.0 ± 1.2
Max - Min T_*max*_ (Change)		28.0–17.0 (11)	23.5–11.0 (12.5)		28.0–22.5 (5.5)	28.0–12.0 (16.0)		32.0–16.0 (16.0)
Max - Min T_*min*_ (Change)		16.0–8.0 (8)	11.5–(-0.5) (12.0)		19.0–10.5 (8.5)	17.0–11.0 (6.0)		18.0–4.0 (14.0)

*Measurements were made in years 2011–2013, where fruit maturation started on 277, 281, and 269 days, respectively. Figure 1 presents the changes in meteorological parameters and the first occurrences of physiological disorders. Means followed with same letter in the same row in each year are not significantly different at *p* ≤ 0.05.*

In 2012, fruit maturation initiated in 281 days, while substantial damages were recorded much earlier; %CR and %RS increased (from 4.0% to 11.1% and from 9.0% to 32.9%, respectively) after a fluctuation in T_*min*_ (varied between 19.0°C and 10.5°C) in 267 days, while there was no considerable changes in %RH or rainfall occurrence. A further deterioration in CR and RS was monitored 21 days later (288 days).

In 2013, fruit maturation started in 269 days, with no CR symptoms, and it was later, in 277 days, when CR (14.9%) and RS (27.6%) symptoms developed, similarly after a sharp drop in T_*min*_ (from 18.0°C to 4.0°C), a steady decrease in T_*max*_ (from 32.0° to 16.0°C), and a fluctuation in %RH (49.3%–83.0%).

The first occurrence of symptoms (285 days in 2011, 267 days in 2012, and 277 days in 2013), were positively correlated with changes in T_*max*_ (5.5–16.0°C) (*r*^2^ = 0.801; y = 0.7529 × + 4.0106), but not in T_*min*_ (8.0–14.0°C) (*r*^2^ = 0.225), and caused a 7.1–14.9% increase in %CR. There were no significant correlations found between changes in RS occurrence and temperature fluctuations.

CR and RS symptoms were almost double in 2011 and 2012 (mean 41.1 and 40.0%, respectively), compared with 2013 (14.9 and 27.6%, respectively), as shown in Table 1. Sun scald symptoms did not change during the measurement periods and records were greater in 2011 and 2012 (mean 22.5%), compared with 2013 (10.0%).

### Effects of Canopy Side on the Occurrence of Physiological Disorders and Yield Parameters

Damages from RS and SS were greater in 2012, compared with 2011 (Table 2). A higher occurrence of CR symptoms was found in fruit from the N-, compared with the S-side, of the canopy (control trees, 74.0% vs. 54.6% in 2011 and 73.8% vs. 47.0%, in 2012, respectively). Similarly, %RS was greater in the N-side of the canopy (control trees, 57.3% vs. 2.1% in 2011, and 41.1% vs. 25.3% in 2012, respectively). On the other hand and as expected, SS symptoms were more pronounced in the S- compared with the N-side of the canopy (control trees, 40.2% vs. 0% in 2011 and 33.0% vs. 13.9% in 2012).

**TABLE 2:** *P* values for the effects of ASA, year, canopy position, and their interactions and mean (± SE) percentage (%) fruit with symptoms of cracking (CR), russeting (RS), and sun scald (SS) harvested from the north (N-) and south (S-) side of canopy or total fruits.

		2011				2012	
				
		Control	ASA1	ASA2	Control	ASA1	ASA2
% CR	S-side	54.6 ± 7.4 B	52.7 ± 3.7	58.1 ± 6.5	47.0 ± 6.4 B	42.9 ± 5.7	48.3 ± 8.8
	N-side	74.0 ± 11.1 A	68.8 ± 12.2	81.4 ± 7.6	73.8 ± 7.0 A	48.5 ± 9.7	54.9 ± 13.5
% RS	S-side	2.1 ± 2.1 B	2.3 ± 2.3 B	0.0 ± 0.0 B	25.3 ± 2.4 aB	17.4 ± 2.7 bB	21.9 ± 5.1 ab
	N-side	57.3 ± 16.7 aA	17.4 ± 5.8 bA	30.0 ± 9.4 abA	41.1 ± 3.6 aA	32.2 ± 5.4 bA	29.8 ± 5.4 ab
% SS	S-side	40.2 ± 5.3 A	32.5 ± 2.6 A	42.1 ± 8.5 A	33.0 ± 2.3 A	26.9 ± 3.2 A	28.6 ± 2.4 A
	N-side	0 ± 0 B	0 ± 0 B	0 ± 0 B	13.9 ± 2.1 B	11.4 ± 2.1 B	20.5 ± 4.8 B
Total% CR		62.0 ± 7.6	55.4 ± 1.6	68.8 ± 5.0	53.5 ± 5.0	45.4 ± 5.9	49.8 ± 9.8
Total% RS		23.3 ± 3.6 a	10.1 ± 3.6 b	22.4 ± 4.7 a	30.2 ± 3.2 a	22.9 ± 3.2 b	24.9 ± 5.0 b
Total% SS		22.8 ± 2.6	19.8 ± 4.9	21.9 ± 4.2	23.3 ± 1.3	21.0 ± 2.6	25.4 ± 2.6

***P* values**	**ASA**	**Year**	**Position**	**ASA × Year**	**ASA × Position**	**Year x Position**	**Year x ASA x Position**

%CR	0.420	0.055	0.003	0.594	0.715	0.493	0.736
%RS	0.001	0.000	0.000	0.071	0.009	0.000	0.006
%SS	0.124	0.000	0.000	0.994	0.613	0.000	0.260

*Trees were sprayed with 0.5 mM, 1.0 mM acetylsalicylic acid (ASA), while control trees were unsprayed. Measurements were made 2 days before the first commercial harvest in 2011 and 2012. Means followed with the same lower case letters are not significantly different at *P* < 0.05 across spraying treatments, and with capital letters across S- and N-side positions in the canopy.*

An increase in yield because of greater fruit numbers was found in the S- compared with the N- side of the canopy; for example, in control trees the yield was 9.3 vs. 3.8 kg, and the fruit number was 22.8 vs. 9.3, respectively (Table 3). There was no significant effect of position in the canopy on fruit fresh weight.

**TABLE 3:** Mean (± SE) yield (kg), fruit number, fruit fresh weight (g), percentage (%) red coloration, peel CIELAB color parameters L*, a*, b*, hue, and Chroma in control (unsprayed) trees or receiving six foliar sprayed applications with 0.5 mM (ASA1) or 1.0 mM (ASA2) acetylsalicylic acid during fruit growth in year 2012.

					*P*		
	
		Control	ASA1	ASA2	ASA	Position	ASA × Position
Yield (Kg)	S-side	9.3 ± 1.7	14.1 ± 3.2	8.7 ± 2.6	0.587	0.006	0.734
	N-side	3.8 ± 1.7	5.1 ± 1.8	3.9 ± 1.8			
# Fruit	S-side	22.8 ± 5.0	32.9 ± 10.0	22.0 ± 5.6	0.618	0.007	0.518
	N-side	9.3 ± 2.6	11.1 ± 4,3	9.3 ± 3.8			
FFW (g)	S-side	415.1 ± 9.0	435.4 ± 10.9	437.5 ± 13.1	0.383	0.700	0.646
	N-side	405.5 ± 13.8	441.0 ± 16.7	431.1 ± 19.5			
Total yield (Kg)		13.1 ± 2.5	19.2 ± 3.8	12.6 ± 2.9	0.326		
Total # Fruit		32.1 ± 6.8	44.0 ± 10.9	29.3 ± 5.9	0.492		
Total FFW (g)		413.0 ± 7.7	435.2 ± 9.3	435.5 ± 9.9	0.101		

*Fruits were separately harvested from the north (N-) and south (S-) side of canopy.*

### Effects of Acetyl Salicylic Acid and Year

The ASA treatment did not significantly affect the presence of CR or SS symptoms (Table 2). Nevertheless, the ASA sprayed trees had usually less fruits with RS symptoms. The 0.5 mM ASA treatment had less RS by 57% in 2011 (from 23.3% to 10.1%), and by 30% in 2012 (from 30.2% to 22.9%) (Supplementary Photo 2). The 1.0 mM ASA treatment reduced RS in total fruit by 18% (from 30.2% to 24.9%) in 2012, whereas there was no significant affect in 2011.

There was no significant effect of ASA treatment on yield, fruit number, or FFW, and there was no significant interaction between ASA and canopy position (Table 3). Nevertheless, there was a trend (*p* = 0.101) of higher FFW in the ASA treated trees compared with control.

**FIGURE 2:**
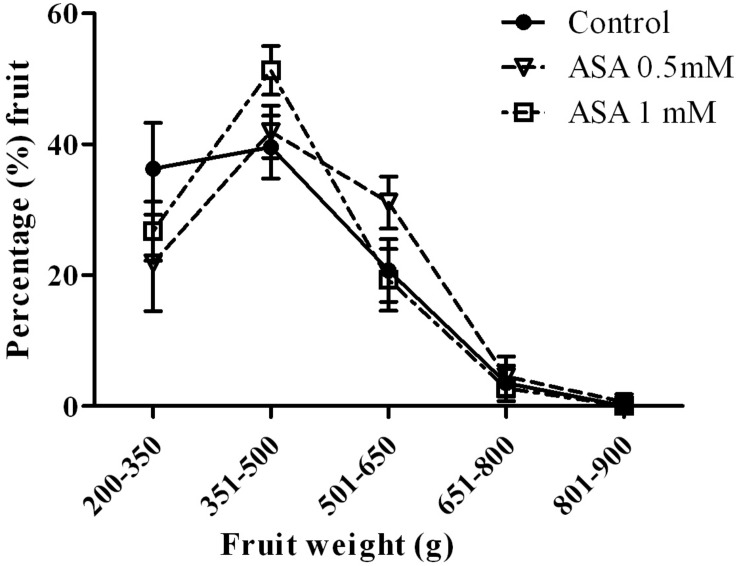
Percentage (%) fruit with different fruit fresh weight categories in trees sprayed with 0.5 or 1.0 mM ASA or unsprayed (control), in year 2012.

Most harvested fruit weighted in the category 351–500 g (43.6%), followed by 200–350 g (29.0%), 500–650 g (23.6%) and less was in the 651–800 (3.6%) and >800 g (0.2%) (Figure 2). There was no significant effect of ASA treatment on the% fruit harvested in different weight categories; nevertheless there was a trend in producing larger sized fruits as suggested by lower% in the smallest size category (*p* = 0.176).

SSC was greater in fruit harvested from trees sprayed with 0.5 mM ASA (mean 17.1 ± 0.3 °Brix), compared with 1.0 mM ASA and the control (mean 16.6 ± 0.2 °Brix) (Table 4). There was no significant effect of ASA treatments of TA and MI. The ASA treatments did not significantly affect the% edible portion (mean 51.4%),% juice (mean 30.9%) and 100 seed weight (mean 36.4 g) (Table 4). Similarly, there was no significant effect of ASA on TPs and ascorbic acid contents, nevertheless, the ASA treatment at 1.5 mM dose increased the TAC_*DPPH*_ in 2011, but not in 2012.

**TABLE 4:** Means (± SE), soluble solid (SSC), (°Brix), total acid (TA), (g citric acid L^–1^), total phenolic (TPs) (mg equivalent gallic acid L^–1^), and ascorbic acid (mg L^–1^) contents and total antioxidant capacity (TAC_*DPPH*_) (mg equivalent ascorbic acid L^–1^), in fruit from pomegranate (cv. Wonderful) trees treated with 0.5 mM (ASA1), 1.0 mM (ASA2) acetylsalicylic acid, or water (control) in 2011 and 2012.

	2011			2012			ANOVA		
			
	Control	ASA1	ASA2	Control	ASA1	ASA2	ASA	Year	Year x ASA
SSC	16.2 ± 0.2	16.7 ± 0.2	16.1 ± 0.3	16.6 ± 0.2	17.1 ± 0.3	16.5 ± 0.2	0.030	0.040	0.996
TA	28.0 ± 1.5	30.1 ± 1.3	30.0 ± 1.3	23.8 ± 1.8	25.1 ± 1.4	28.3 ± 1.1	0.182	0.011	0.513
MI	5.9 ± 0.3	5.6 0.3	6.5 ± 0.3	7.2 ± 0.5	6.9 ± 0.3	5.9 ± 0.3	0.072	0.001	0.335
TPs	2,427.8 ± 52.7	2,488.0 ± 58.0	2,452.8 ± 91.7	1,897.0 ± 42.7	1,830.7 ± 78.2	1,905.2 ± 96.5	0.959	<0.001	0.661
TAC_*DPPH*_	2,450.4 ± 28.8 a	2,606.2 ± 59.4 b	2,434.3 ± 68.7 ab	2,607.6 ± 36.1	2,801.2 ± 30.4	2,734.3 ± 29.7	0.475	0.189	0.007
Ascorbic acid	21.4 ± 0.7	22.7 ± 0.9	20.6 ± 1.5	18.7 ± 0.6	19.5 ± 1.2	19.0 ± 0.7	0.401	0.005	0.716
% edible portion	46.0 ± 0.7	48.1 ± 0.5	48.2 ± 0.7	49.0 ± 1.9	48.7 ± 3.7	47.6 ± 4.2	0.880	0.512	0.601
% juice	31.5 ± 0.7	33.2 ± 0.6	31.3 ± 0.5	34.3 ± 1.5	33.7 ± 2.6	33.0 ± 3.2	0.657	0.148	0.708
100 seed weight	38.1 ± 0.7	37.1 ± 1.0	36.9 ± 0.9	35.5 ± 0.8	35.5 ± 0.7	35.3 ± 0.9	0.773	0.054	0.830
% red				35.0 ± 4.0 b	65.0 ± 3.8 a	67.0 ± 6.0a	<0.001		
L*				55.1 ± 1.1 a	47.1 ± 0.8 b	45.6 ± 1.2 b	<0.001		
a*				43.6 ± 1.2 b	50.2 ± 1.0 a	51.2 ± 1.1 a	<0.001		
b*				21.7 ± 0.3	22.4 ± 0.5	22.6 ± 0.7	0.220		
H*				26.6 ± 0.4 a	22.9 ± 0.4 b	23.8 ± 0.7 b	<0.001		
C*				48.7 ± 1.0 b	54.2 ± 0.6 a	56.0 ± 0.9 a	<0.001		

*ANOVA results from the effects of year and ASA treatment and their interaction are presented. Means with different letters are significantly different at *P* < 0.05 in the same year. The asteristics is part of CIELAB color parameter identifications.*

Percentage (%) red coloration was almost double in 0.5 mM and 1.0 mM ASA (mean 66.0% ± 5.0), compared with the control (35.0% ± 4.0) (Table 4 and Supplementary Photo 2). The fruits harvested from trees treated with ASA had greater *a*^∗^ (red coloration) and *C*^∗^ values, lower *L*^∗^ and *H*^∗^, whereas there was no effect on *b*^∗^.

Fruits harvested in 2012 was at a more advanced maturing stage as suggested by a higher SSC (16.7 vs. 16.3) and MI (6.7 vs. 5.7), lower TA (25.7 vs. 29.4), and also had lower TPs (1,877.7 vs. 2,456.2) and ascorbic acid (19.1 vs. 21.6) contents. There was no significant effect of year on TAC_*DPPH*_,% edible portion,% juice, and 100 seed weight.

**FIGURE 3:**
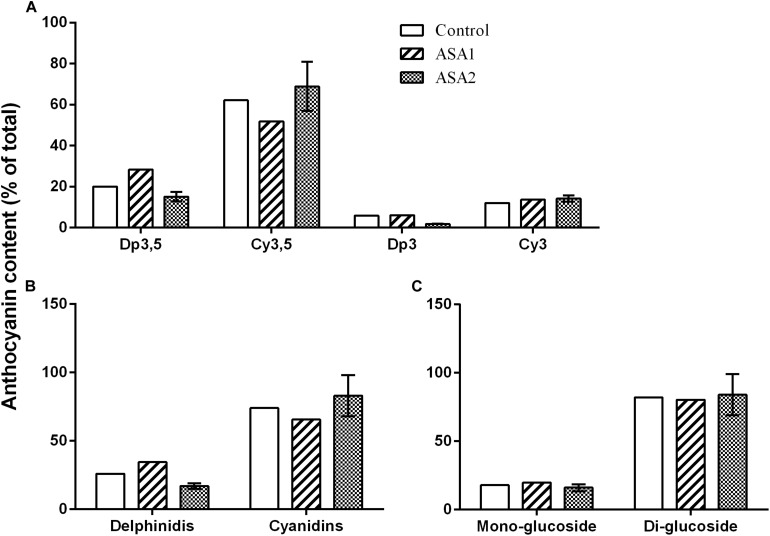
Effects of foliar applications with 0.5 mM (ASA1) or 1.0 mM (ASA2) acetyl salicylic acid on the relative abundance of **(A)** individual anthocyanins, **(B)** delphinidins and cyanidins, and **(C)** mono- and diglucosides in the pomegranate juice.

**FIGURE 4:**
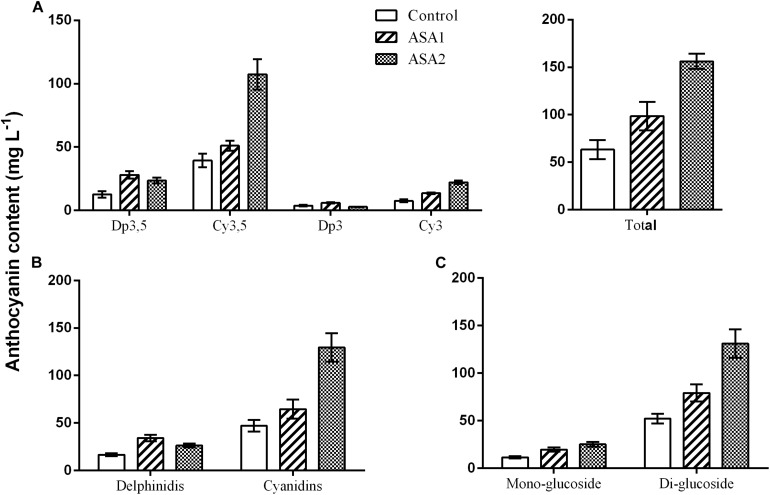
Effects of foliar applications with 0.5 mM (ASA1) or 1.0 mM (ASA2) acetyl salicylic acid on the concentration of **(A)** the total and individual anthocyanins, **(B)** delphinidins and cyanidins, and **(C)** mono- and diglucosides in the pomegranate juice.

Anthocyanins consisted mainly of Cy3,5 (62.1% of total) followed by Dp3,5 (20.0% of total) and lower amounts of Cy3 (12.0% of total), and Dp3 (5.9% of total) (Figure 3A). Most anthocyanins were in the form of cyanidins and less as delphinidis (Figure 3B) while more was as di-glucosides and less as mono-glucosides (Figure 3C). The ASA treatment did not alter the relative abundance of anthocyanins (Figure 3). Nevertheless, there was an increase in the juice anthocyanin concentration especially in the 1.0 mM ASA and less in the 0.5 mM ASA, compared with control (Figure 4). ASA2 induced a more pronounced increase in Cy3,5 compared with the rest measured anthocyanins and in cyanidins compared with delphinidis (Figures 4A,B).

Organic acid and mineral nutrient contents were not affected by the ASA treatments (data not shown): citric acid (mean 29.6 ± 0.5 g L^–1^), electric acid (mean 0.6 ± 0.1 g L^–1^) and ellagic acid (mean 1.2 ± 0.2 mg L^–1^). Minerals constituents was potassium, magnesium, calcium, and sodium at a concentration of 1,866.7, 63.0, 52.0, and 37.0 mg L^–^^1^, respectively.

## Discussion

The fruit maturation periods, during the present 3 year study, were characterized with few rainfalls, which were not related with CR damages. Instead, the first occurrence of CR symptoms, were attributed mainly to fluctuations in T_*max*_ (5.5–16.0°C) (*r*^2^ = 0.801; y = 0.7529 × + 4.0106) and less in T_*min*_ (8.0–14.0°C) (*r*^2^ = 0.225), causing a 7–15% increase in %CR. The pomegranate fruit has thick peel, and it is probable that the pulp and the peel shrink due to temperature difference in a disanalogous proportion and subsequently crack. At a later maturity stage (298 days in 2011 and 288 days in 2012) damages were further accelerated; in 2011 this probably resulted from freezing damage, as temperature dropped to −0.5°C, related with mechanical effects of ice formation within tissues (Burke et al., 1976). To our knowledge, this is the first study that documents relations between fluctuations in meteorological parameters, other than rainfall on the occurrence of CR in pomegranate (Galindo et al., 2014) while changing conditions of temperature or vapor pressure deficit were implicated with CR in tomato (Guichard et al., 2001). In the present experimental orchard, RS damages were extensive, and this was also the case in nearby orchards of cv. “Wonderful,” but not in cv. “Acco,” suggesting that its occurrence is cultivar dependent and favored by local environmental conditions. Similarly to CR damage, RS was induced by temperature fluctuation as suggested by the fact that occurrence was monitored after a change in T_*min*_ or T_*max*_ in all studied years. The development of RS damage may also be related with increased RH as suggested by the fact that symptoms were more pronounced in (i) years with higher RH (2011 and 2012 vs. 2013), and (ii) in the N- compared with the S-part of canopy, where higher RH would be expected. Moreover symptoms often occurred at the stylar end, where free water may accumulate and were also exacerbated in the skin of fruits covered with paper or plastic bag, compared with unbagged fruits in trees from the present experimental orchard (unpublished data). Similar findings relating RS development with increased RH or water movement were reported in plums (Michailides, 1991) and apples (Tukey, 1959; Knoche and Grimm, 2008). Further studies are required to elaborate the mechanism and causal agents for inducing RS damages in cv. “Wonderful.”

Effects of row orientation on performance of fruit trees trained in hedgerows or other designs are poorly investigated, although a N–S orientation is preferred (Trentacostea et al., 2015). In the present study the experimental orchard was planted in a W–E orientation, which proved to be detrimental for fruit quality as depicted by the presence of substantially higher CR and RS symptoms in the N- compared with the S-, side of the canopy. Conventional wisdom concludes that N-S row orientation leads to lower temperature and less light in the N-side, which may have caused differences in the fruits’ cuticle thickness. A thicker inelastic cuticle is expected to be more prone to CR and was shown to develop in shaded apples increasing their sensitivity of CR (Shutak and Schrader, 1948). Moreover, oranges grown on the shaded part of the tree had a significantly greater percentage of fruit cracking (Li and Chen, 2017).

Moreover, the N-side of the canopy had lower fruit number and yield, without any effect on FFW. It is probable that shading negatively affected flower formation and/or fruit set as this has been reported in other horticultural species (Marini and Sowers, 1990; Hampson et al., 1996; Ferree et al., 2001). Similarly, in a nearby bush trained pomegranate orchard, where inner sprouting was un-removed, the inner part of trees was usually without fruit, while RS symptoms were more abundant. Therefore, it is important to avoid planting in a W-E orientation especially in northern Greece while this may be less critical in southern locations of the tropical and subtropical regions that the majority of pomegranate is grown worldwide.

In the present study spraying 4 or 6 times before harvest at 0.5 mM or 1.0 mM ASA did not alter the presence of CR and SS symptoms. Instead, the ASA sprayings substantially alleviated RS damages by up to 57%. Moreover the peel coloration improved in fruit from the ASA treated trees, as shown by an increase in the% red coloration, *a*^∗^ and *C*^∗^ values and lower *L*^∗^ and *H*^∗^ values. We are not aware of another study on the effects of salicylic acid on russeting in apple, pear, or plums. Previous studies have shown a beneficial role of salicylic acid or its derivatives when applied at pre- or postharvest stage in physiological disorders developing during storage, such as scald in apple (Giannousis, 2012) and reducing chilling injury (CI), and internal browning in pomegranates (Sayyari et al., 2009, 2011a,b; Dokhanieh et al., 2016) and peaches (Wang et al., 2006). In the present study RS damages were apparent in fruits from cv “Wonderful” but not cv. “Acco.” In the latter cultivar, the skin has a better coloration being fully red colored and perhaps the beneficial effects of ASA on RS symptoms in cv “Wonderful” may be related with effects on red pigmentation in the skin tissue.

Anthocyanins contribute to the red/purple color of pomegranates in peel or juice and the predominant class of anthocyanins recovered in pomegranate juice belongs to the cyaniding, compared with the delphinidin and pelargonidin groups (Beaulieu et al., 2015), which were also the case in the present study. The Dp3,5 and Cy3,5 contents were greater compared with the Dp3 and Cy3 contents. An increase in the juice anthocyanin concentration especially in the 1.0 mM ASA and less in the 0.5 mM ASA compared with the control was found in the present study, while1.0 mM ASA induced a more pronounced increase in Cy3,5 compared with the rest measured anthocyanins and in cyanidins compared with delphinidis. ASA sprayings also improved TAC_*DPPH*_ and SSC contents in juice. The above beneficial effects of ASA on fruit quality attributed were also documented in other studies on pomegranate (Sayyari et al., 2011b; Sayyari and Valero, 2012; García-Pastor et al., 2020a) or other fruit species (apples, Giannousis, 2012; pears, Cao et al., 2006(sweet cherries, Yao and Tian, 2005; Giménez et al., 2014, 2017; Valverde et al., 2015; oranges, Huang et al., 2008(grapes, Oraei et al., 2019; García-Pastor et al., 2020b; plums, Martínez-Esplá et al., 2017, 2018). The pomegranate cv. “Wonderful” is known to have high antioxidant contents in juice, compared with other cultivars (Pantelidis et al., 2012; Chater et al., 2018), and therefore, a further increase by foliar sprays with ASA may be less important from a commercial point of view, compared with the improvement in the external fruit appearance such as lowering RS symptoms and better red coloration documented in the present study (Supplementary Photo 2).

There were no substantial effects of ASA treatment on yield-related parameters; however, there was a trend in producing heavier fruit as suggested by an increase in total FFW, decrease in the %fruit in 200–350 g (smaller size) and increase in the %fruit of 351–500 g and 501–650 g size categories. There were no effects of ASA treatment on% edible portion,% juice and 100 seed weight. As expected the harvested fruit number was not affected by the ASA treatment, since the foliar applications started after 2 or 3 months of fruit development (August 19, 2011 and July 17, 2012). Similar to our findings on increasing fruit yield-related attributes on pomegranate were results on sweet cherry (Giménez et al., 2014), plum (Martínez-Esplá et al., 2017, 2018), grapes (Marzouk and Kassem, 2011; Champa et al., 2015; García-Pastor et al., 2020b), and apples (Shaaban et al., 2011). Increases in productivity by salicylic acid or its derivatives such as ASA were previously attributed to enhanced leaf area, photosynthetic pigments concentration in leaves, photosynthetic rate, and translocation of sugars from leaves to fruit; however, a controversial role on plant growth depending on cultivar, its concentration, plant growth conditions, and developmental stages has been reported (Rivas-San Vicente and Plasencia, 2011).

## Conclusion

The present study reports for the first time that pomegranate CR and RS were induced by temperature fluctuations during the fruit maturation period, and this parameter needs to be considered before establishing an orchard where temperature usually drop during the fruit maturation period. Extensive damages from RS were also reported in the present study that was further related with increased RH and water accumulation. Planting in a W–E direction should be avoided at least in higher latitudes as in the N- compared with the S- side of the canopy, CR and RS symptoms were greater, and fruit yield was lower. ASA is a natural and safe compound, and foliar sprays at 0.5 or 1.0 mM proved to have beneficial effects on juice antioxidant contents, but more importantly on fruit appearance, they are shown as a reduction of RS symptoms and color improvement.

## Data Availability Statement

The original contributions presented in the study are included in the article/Supplementary Material, further inquiries can be directed to the corresponding author/s.

## Author Contributions

PD conceived the project and wrote the manuscript. GP and PD designed the experiment and executed the field and laboratory work. SV undertook the anthocyanin, organic acid, and mineral content analyses. All authors have read and approved the final manuscript.

## Conflict of Interest

The authors declare that the research was conducted in the absence of any commercial or financial relationships that could be construed as a potential conflict of interest.
