# Supplementary material for: Physiological Disorders and Fruit Quality Attributes in Pomegranate: Effects of Meteorological Parameters, Canopy Position and Acetylsalicylic Acid Foliar Sprays
Source: Front Plant Sci. 2021 Mar 11;12:645547. doi: 10.3389/fpls.2021.645547 (PMC7991580; doi:10.3389/fpls.2021.645547)
Supplement: Supplementary file 1 [file Data_Sheet_1.docx]

Physiological disorders and fruit quality attributes in pomegranate: Effects of meteorological parameters, canopy position and acetylsalicylic acid foliar sprays

Pavlina Drogoudi^1,*^, Georgios E. Pantelidis^1^, Stavroula A. Vekiari^2^

^1^Hellenic Agricultural Organization (HAO) 'Demeter', Institute of Plant Breeding and Genetic Resources, Department of Deciduous Fruit Trees, 38 R.R. Station, 59035 Naoussa, Greece

^2^Institute of Technology of Agricultural Products, HAO ‘Demeter’, 1 S. Venizelou, Lykovrissi, 14123 Athens, Greece

*** Correspondence:**Corresponding Author
[drogoudi@otenet.gr](mailto:drogoudi@otenet.gr)

Keywords: anthocyanins; antioxidants; cracking; fruit weight and number; russeting; sun scald; total soluble content; yield

Words: 6315

Tables: 4

Figures 4

Photo: 1


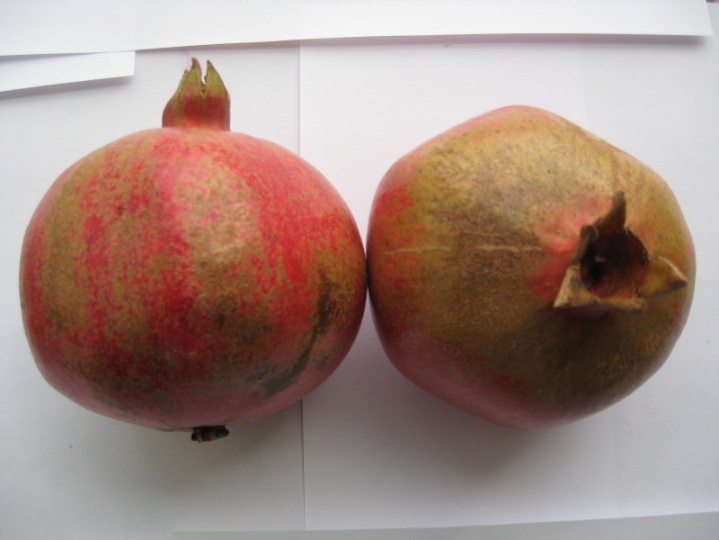


**Supplementary Photo 1.** Symptoms of russeting in pomegranate fruit cv. ‘Wonderful’.

| **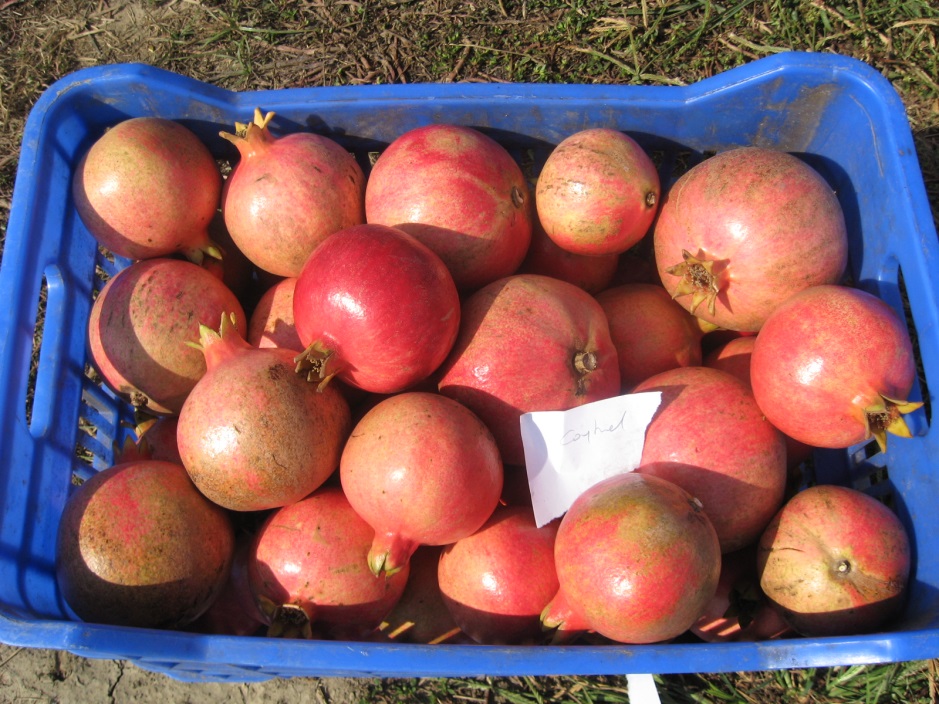A** | **B** |
| --- | --- |

**
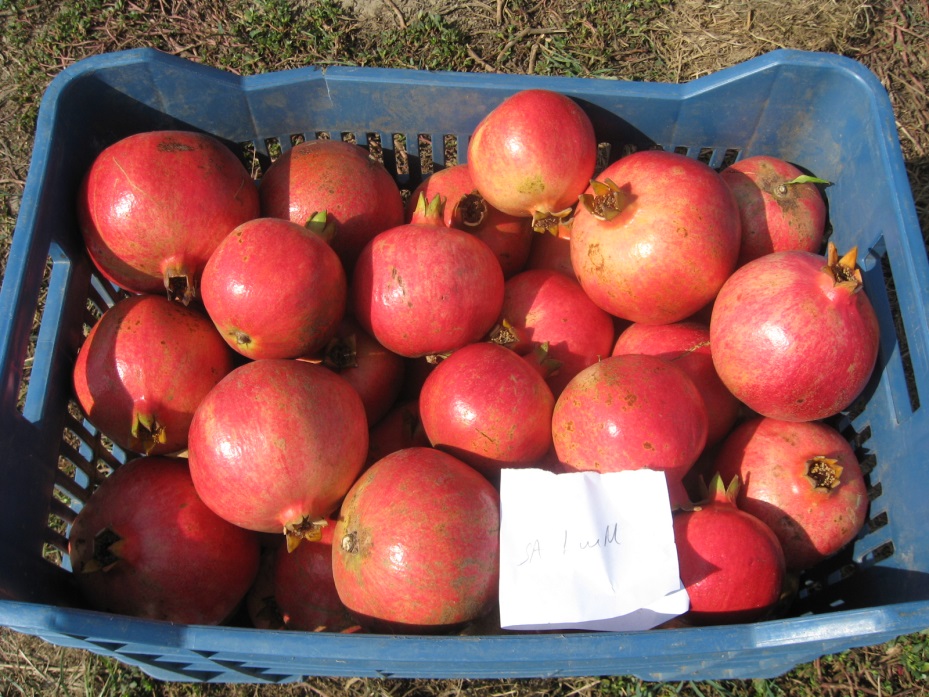
**

**Supplementary Photo 2. A**ppearance of harvested pomegranates from trees (A) unsprayed (control), and (B) sprayed with ASA 1 mM; the latter showing less russeting and better red coloration.
